# Association of Refractive Error Severity, Axial Length and Digital Screen Time with Dry Eye Disease in Young Saudi Adults

**DOI:** 10.3390/jcm15197358

**Published:** 2026-09-22

**Authors:** Wafa Majed Alotaibi, Maha Naif, Muhammad Qasim, Anas S. Alakhras, Muteb Alanazi, Mosaad Alhassan, Ali Almustanyir, Balsam Alabdulkader, Meznah Almutairi, Mohammed Althomali, Mohammed Alhazmi, Tahani Alqahtani

**Affiliations:** 1 Optometry Department, College of Applied Medical Sciences, King Saud University, Riyadh 12372, Saudi Arabia; 443200380@student.ksu.edu.sa (M.N.); mkalanazi@ksu.edu.sa (M.A.); malhassan@ksu.edu.sa (M.A.); aalmustanyir@ksu.edu.sa (A.A.); balabdulkader@ksu.edu.sa (B.A.); mzalmutairi@ksu.edu.sa (M.A.); malthomali@ksu.edu.sa (M.A.); malhazmyi@ksu.edu.sa (M.A.); talqahtani@ksu.edu.sa (T.A.); 2 Department of Optometry and Vision Sciences, School of Sciences, University of Minho Gualtar Campus, 4710-057 Braga, Portugal; 3 Vocational Training Corporation, Amman 11190, Jordan; anas.alakhras@gmail.com

**Keywords:** dry eye, refractive error, axial length, myopic, hyperopic, OSDI, TBUT, NI-TBUT

## Abstract

**Background:** Dry eye disease (DED) and refractive errors are increasingly prevalent among young adults, particularly in populations with prolonged digital device use. However, the relationship between refractive error, axial length (AL), and dry eye parameters remains incompletely understood. **Methods:** A cross-sectional observational study was conducted among 114 young Saudi adults attending the optometry clinic of King Saud University. Participants underwent comprehensive ocular examination including refractive assessment, AL measurement, Ocular Surface Disease Index (OSDI), non-invasive tear breakup time (NI-TBUT) and tear breakup time (TBUT). Refractive error was classified into hyperopia and myopia, with further stratification by severity. Continuous variables are presented as mean ± standard deviation (SD), while categorical variables are summarized as frequencies and percentages. One-way ANOVA was performed among refractive error groups, whereas the Kruskal–Wallis H test was used for dry eye severity. Pearson’s correlation, Spearman’s correlation, Bonferroni-adjusted post-hoc analysis, and linear regression analysis were performed, keeping the *p*-value < 0.05. The study got IRB approval and adhered to the tenets of the declaration of Helsinki. **Results:** The mean age of participants was 23.95 ± 3.73 years, with 64.9% females. Mean daily screen-time was 9.40 ± 2.93 h. Axial length demonstrated a weak positive correlation with OSDI scores; however, it did not reach statistical significance (r = 0.174, *p* = 0.065). Similarly, AL was not significantly correlated with TBUT (r = −0.134, *p* = 0.157) or NI-TBUT (r = −0.147, *p* = 0.120). In contrast, screen time showed significant correlations with the OSDI scores (r = 0.650, *p* < 0.001), TBUT (r = −0.585, *p* < 0.001), and NI-TBUT (r = −0.509, *p* < 0.001). Dry eye severity differed significantly across refractive error groups (Kruskal–Wallis H = 18.762, *p* = 0.002), showing higher refractive error associated with greater dry eye severity. Multivariable regression analyses demonstrated that greater screen time was significantly associated with higher OSDI scores and shorter TBUT and NI-TBUT, whereas spherical equivalent (SE) was not independently associated with any of these dry eye outcomes after adjusting for demographic variables. Additionally, greater screen time was significantly associated with composite dry eye severity (Spearman’s ρ = 0.639, *p* < 0.001). **Conclusions:** Prolonged digital screen exposure showed the most consistent association with greater dry eye severity, whereas refractive error and axial length were not independently associated with dry eye outcomes after adjustment for screen time and demographic factors. Although unadjusted differences exist across certain refractive subgroups, behavioral factors such as screen time may contribute to dry eyes. These findings support comprehensive dry eye evaluation in individuals with refractive error and prolonged digital device use.

## 1. Introduction

Dry eye disease (DED) is a common multifactorial ocular surface disorder characterized by loss of tear film homeostasis and associated with ocular symptoms, tear film instability, hyperosmolarity, ocular surface inflammation, and neurosensory abnormalities [1,2]. Maintaining tear film stability is essential for ocular comfort and optical quality, and DED is increasingly recognized among younger adults [1]. Both subjective symptoms and objective tear film abnormalities may occur, highlighting the importance of combining patient-reported outcomes with objective clinical measurements when evaluating DED [3,4].

Refractive status and ocular biometric characteristics may be associated with ocular surface health. Myopia is commonly accompanied by axial elongation, and recent evidence suggests an association between greater AL and dry eye-related abnormalities. Huang et al. reported higher OSDI symptom scores among myopic children compared with non-myopic children [5]. In adults with myopia, Lyu et al. reported that greater AL was associated with increased prevalence and severity of ocular surface abnormalities, including shorter tear film breakup time and higher OSDI scores [6]. Similarly, Ayaki et al. identified a sex-specific association between AL and tear production in elderly patients, suggesting that ocular biometric characteristics may be relevant to tear film function [7]. However, these studies have largely focused on specific age groups or myopic populations, limiting understanding of the relationship across different refractive error categories in young adults.

Evidence from Saudi Arabia is also limited. Fahmy and Aldarwesh investigated the relationship between refractive error and dry eye in Saudi young adults using non-invasive Keratograph assessment [8]. Although this study has provided important regional evidence, a further investigation is warranted using a broader assessment of refractive status, together with AL and both subjective and objective dry eye measures. In particular, limited evidence is available regarding whether dry eye manifestations differ across varying degrees of refractive error, including hyperopia and high myopia, in young Saudi adults.

Digital screen exposure represents an additional potentially important risk factor for DED in young adults. Prolonged digital device use has been associated with reduced or incomplete blinking and increased ocular surface symptoms [9,10]. In Saudi university students, Albalawi et al. reported an association between screen time, sleep quality, and dry eye symptoms; however, their assessment relied primarily on subjective measures [11]. Zarban et al. similarly reported a high prevalence of dry eye symptoms among university students in Western Saudi Arabia in association with electronic device use [12]. These findings support the relevance of digital exposure in young adults, but the relationship between screen exposure, ocular biometry, refractive status, and objectively measured tear film stability remains insufficiently characterized.

Therefore, this cross-sectional study aimed to investigate the differences in dry eye parameters across different refractive error categories among young Saudi adults. Specifically, we compared subjective dry eye symptoms, assessed using the Ocular Surface Disease Index (OSDI), and objective tear film parameters, including non-invasive tear breakup time (NI-TBUT) and tear film breakup time (TBUT) across different refractive error groups. We also evaluated the associations of spherical equivalent (SE) and AL and digital screen exposure with dry eye parameters. By integrating refractive status, AL, digital screen exposure, and both subjective and objective measures of DED, this study seeks to provide a more comprehensive assessment of the factors associated with dry eye in young Saudi adults.

## 2. Materials and Methods

This cross-sectional observational study was conducted at the optometry clinic of the College of Applied Medical Sciences (CAMS) at King Saud University in Riyadh, Saudi Arabia, from January to May 2026. The study adhered to the Declaration of Helsinki, and received institutional review board (IRB) approval with reference number ES-25-10339 dated 4 December 2025 from the committee of Health Sciences Colleges Research on Human Subjects, CAMS, King Saud University, Riyadh, Kingdom of Saudi Arabia. Informed written consent was taken from each participant included in this study. A priori sample size calculation was performed using G*Power version 3.1.9.7 for multiple linear regression (fixed model, R^2^ deviation from zero). Assuming a medium effect size (f^2^ = 0.15), two predictors (spherical equivalent and screen time), a two-sided significance level of 0.05, and 80% statistical power, the minimum required sample size was determined as 68 participants. The study enrolled 114 healthy Saudi young adults aged between 19 and 35 years from a single optometry clinic using the convenient sampling technique. Although ocular examinations were performed on both eyes, only data obtained from the right eye were included in the statistical analyses to avoid potential bias resulting from the correlation between fellow eyes, and to ensure the statistical independence of the observations. The inclusion criteria required a complete medical history and subjects having refractive errors > ±0.50, while the exclusion criteria involved the presence of systemic conditions (e.g., Sjögren’s syndrome, hypertension, and diabetes), a history of ocular surgery, contact lens wear, smoking, and regular medication use.

Participants were categorized into groups based on myopic and hyperopic refractive severity levels (low, moderate, high/severe) calculated from the spherical equivalent (SE), as shown in Table 1 [13,14].

DED was assessed using subjective symptoms (OSDI) and objective tear film parameters (NI-TBUT and TBUT), interpreted according to predefined study-specific clinical categories, as shown in Table 2 [15,16,17,18,19,20,21,22].

Dry eye severity was classified based on three parameters, i.e., NITBU, TBUT and OSDI. TBUT and NITBUT were considered the primary objective measures, whereas OSDI served as a supportive symptom-based measure. An overall severity category was assigned when at least two of the three parameters were classified within the same severity category. In the event of discordance among the three parameters, the more severe category was predefined as the final classification; however, no participant in the present study required the application of this rule. The right eye was selected as the study eye for all participants so as to ensure a standardized and consistent unit of analysis.

### 2.1. Study Protocol

Upon arrival, demographic details and daily average screen time (including smartphone, computer, television, etc.) were collected on the designed proforma. To maintain consistency, participants refrained from caffeine before the visit, and a uniform examination sequence was followed for both eyes separately, allowing adequate rest to avoid fatigue. Testing proceeded in the clinical environment with adequate room temperature during daytime and followed the informed written consent. The stepwise study protocol is presented stepwise, as shown in Figure 1 for all the participants enrolled in this study. The OSDI Questionnaire [23] was administered first to measure subjective symptoms, visual disturbances, and environmental triggers.

Refractive status was determined using an automated refractokeratometer (Nidek ARK-1, Nidek Co., Ltd., Gamagori, Aichi, Japan) to obtain spherical and cylindrical refractive values. The spherical equivalent (SE) was then calculated and used to classify myopic and hyperopic categories based on severity. Cycloplegic refraction was not performed because cycloplegic agents can temporarily alter tear film characteristics and ocular surface physiology, potentially affecting the evaluation of dry eye.

Axial length was measured using the Pentacam AXL (Oculus Optikgeräte GmbH, Wetzlar, Germany). Participants were instructed to fixate steadily on the instrument’s internal target. The average of three readings was used as the final AL measurement for each participant to enhance measurement precision and reproducibility.

NI-TBUT was then measured using the OCULUS Keratograph 4 (Type 70670, Oculus Optikgeräte GmbH, Wetzlar, Germany) to assesses tear film stability by monitoring the distortion of reflected Placido rings without the need for fluorescein dye. TBUT was evaluated using a slit-lamp biomicroscope (Topcon DC-3, Topcon Corporation, Tokyo, Japan) equipped with a cobalt blue filter. A fluorescein sodium strip (Entod Research Cell UK Ltd., distributed by Haag-Streit AG, Köniz, Switzerland) was gently applied to the lower conjunctival fornix after moistening with sterile saline. Participants were instructed to blink naturally several times before keeping their eyes open, and the interval from the final blink to the first appearance of a dry spot on the cornea was recorded.

### 2.2. Statistical Analysis

Statistical analyses were performed in Microsoft Excel for data organization and IBM SPSS (Version 26). Continuous variables were assessed for normality using the Shapiro–Wilk test and are presented as mean ± standard deviation (SD), while categorical variables are summarized as frequencies and percentages. Normality was assessed through the visual inspection of Q–Q plots. Statistical tests were selected according to the distributional characteristics of the data and the assumptions of each analysis, whereas the distribution of dry eye severity among refractive error groups was compared using the Kruskal–Wallis H test. When overall tests were statistically significant, Bonferroni-adjusted post-hoc pairwise comparisons were performed. Pearson’s correlation analysis was used to assess the associations between continuous variables, whereas Spearman’s rank correlation was used for ordinal variables. Associations of SE, AL and screen time with continuous dryness metrics were evaluated using multiple linear regression, with collinearity evaluated using variance inflation factors (VIF). Statistical significance was set as a two-sided *p* value < 0.05.

## 3. Results

### 3.1. Baseline Demographic Characteristics of the Study Cohort

The study evaluated a total of 114 participants (myopic = 63, hyperopic = 51) with a mean age of 23.95 ± 3.73 years, presenting a gender distribution of 64.9% females (n = 74) and 35.1% males (n = 40). Table 3 presents the descriptive analysis for the continuous and categorical variables established the baseline metrics for the cohort, noting a mean daily screen time of 9.40 ± 2.93 h, a mean AL of 23.68 ± 2.70 mm, and a mean spherical equivalent (SE) of −0.88 ± 4.34 D. Dry eye parameters showed a mean OSDI score of 35.85 ± 18.25, a mean TBUT of 5.34 ± 2.76 s, and a mean NI-TBUT of 6.68 ± 3.33 s.

Table 4 presents the distribution of dry eye disease severity among the 114 study participants. More than half of the participants (60 participants, 52.6%) were classified as having severe dry eye according to the study-specific composite classification; moderate dry eye disease was identified as 25 participants (21.9%), while mild dry eye was observed in 20 participants (17.5%). Only nine participants (7.9%) had a normal ocular surface. Overall, 92.1% of the study participants met the predefined study classification for some degree of dry eye severity based on the assessed subjective and objective parameters.

As shown in Table 5, significant differences among refractive error groups were observed for OSDI and NI-TBUT (one-way ANOVA, *p* = 0.003 and *p* = 0.005, respectively). For TBUT, Levene’s test indicated heterogeneity of variance (*p* = 0.019); therefore, Welch’s ANOVA was used and did not demonstrate a statistically significant difference among groups (Welch F(5,49.202) = 2.110, *p* = 0.080). Bonferroni-adjusted post-hoc comparisons were therefore performed only for outcomes with significant overall group differences. The high myopic group had the highest OSDI score (48.58 ± 18.42), the shortest TBUT (3.93 ± 2.46 s) and the lowest NI-TBUT (4.78 ± 2.65 s), followed by moderate hyperopic (OSDI 40.13 ± 13.64, TBUT 4.68 ± 1.76) and high hyperopic (NI-TBUT 5.96 ± 2.71). In contrast, the low hyperopic group demonstrated the lowest OSDI (27.25 ± 16.98), longest TBUT (6.55 ± 3.34 s) and highest NI-TBUT (8.90 ± 3.50 s) reflecting better ocular surface health. Bonferroni-adjusted post-hoc comparisons for subjective and objective dry eye parameters across refractive error groups showed that for OSDI, participants with high myopic categorization demonstrated significantly higher OSDI scores than those with low hyperopic (mean difference = −21.33, 95% CI: −37.89 to −4.77, *p* = 0.003) and low myopic (mean difference = −18.54, 95% CI: −34.56 to −2.51, *p* = 0.011) categorizations, indicating more severe subjective dry eye symptoms in the high myopic group. A comparable pattern was observed for the NI-TBUT, where the high myopic group exhibited significantly lower values than the low hyperopic group (mean difference = 4.11 s, 95% CI: 1.08–7.14, *p* = 0.001). These findings indicate that individuals with high myopia experienced significantly greater subjective dry eye symptoms and lower NI-TBUT values than participants with lower degrees of refractive error. In contrast, the overall difference in TBUT was not statistically significant when assessed using Welch’s ANOVA.

### 3.2. Correlation Between Axial Length, Screen Time and Dry Eye Parameters

**Axial Length:** Pearson’s correlation analysis showed a weak positive correlation between AL and OSDI scores; however, this association was not statistically significant (r = 0.174, *p* = 0.065). Similarly, AL was not significantly correlated with the objective tear film measures TBUT (r = −0.134, *p* = 0.157) or NI-TBUT (r = −0.147, *p* = 0.120). In contrast, strong negative correlations were observed between OSDI scores and both TBUT (r = −0.824, *p* < 0.001) and NI-TBUT (r = −0.737, *p* < 0.001), indicating that lower tear film stability was associated with greater self-reported dry eye symptoms (Figure 2).

**Screen Time:** Screen time showed statistically significant associations with all assessed dry eye parameters. A strong positive correlation was observed between screen time and OSDI scores (r = 0.650, *p* < 0.001), while significant negative correlations were observed with TBUT (r = −0.585, *p* < 0.001) and NI-TBUT (r = −0.509, *p* < 0.001). These findings indicate that greater screen time was associated with higher self-reported dry eye symptoms and reduced tear film stability (Figure 2). 

An ordinal logistic regression model was initially considered to examine the association between screen time and dry eye parameters; however, the proportional odds assumption was violated. Therefore, Spearman’s rank correlation was used to assess the association between screen time and dry eye parameters. A statistically significant positive correlation was observed between screen time and dry eye parameters (Spearman’s ρ = 0.639, *p* < 0.001, N = 114), indicating that greater daily screen time was associated with higher dry eye level, as can be seen in the Supplementary Data Tables S1–S7.

### 3.3. Dryness Severity Among Refractive Error Groups

To evaluate differences in study-specific dry eye severity across the refractive error groups, a Kruskal–Wallis H test was conducted (Figure 3). The analysis revealed a statistically significant difference in the distribution of dryness severity across the six refractive error groups (χ^2^(5) = 18.762, *p* = 0.002, N = 114). The Bonferroni-adjusted post-hoc pairwise comparisons indicate that participants in the high myopia group had significantly higher dryness severity compared with both the low hyperopia group (adjusted *p* = 0.005, test statistic = −34.954) and the low myopia group (adjusted *p* = 0.011, test statistic = −31.601). This indicates an increasing trend whereby the advancing of refractive error is associated with an upward shift in dry eye symptom severity. These results indicate localized differences in unadjusted dry eye severity across specific refractive groups, driven primarily by higher severity in the high myopia subgroup relative to lower refractive error subgroups, rather than a single continuous trend across all groups.

### 3.4. Association of Screen Time and Spherical Equivalent with Dry Eye Parameters

Table 6 presents multivariable linear regression models evaluating the associations of screen time and SE with dry eye parameters. All three linear regression models were statistically significant (all *p* < 0.001). The OSDI model explained 42.6% of the variance in OSDI scores (R^2^ = 0.426; adjusted R^2^ = 0.416; F(2,111) = 41.186), while the TBUT and NI-TBUT models explained 34.2% (R^2^ = 0.342; adjusted R^2^ = 0.330; F(2,111) = 28.884) and 26.1% (R^2^ = 0.261; adjusted R^2^ = 0.248; F(2,111) = 19.641) of the variance, respectively.

Screen time was significantly associated with all three dry eye parameters. Greater screen time was associated with higher OSDI scores (B = 3.980, SE = 0.460, β = 0.638, 95% CI: 3.068–4.892, *p* < 0.001), indicating greater dry eye symptoms. In contrast, screen time was negatively associated with both TBUT (B = −0.548, SE = 0.074, β = −0.581, 95% CI: −0.695 to −0.400, *p* < 0.001) and NI-TBUT (B = −0.567, SE = 0.095, β = −0.498, 95% CI: −0.756 to −0.379, *p* < 0.001), indicating shorter tear film stability with increasing screen time.

Spherical equivalent was not significantly associated with OSDI score (B = −0.234, SE = 0.310, β = −0.056, 95% CI: −0.848–0.381, *p* = 0.453), TBUT (B = 0.010, SE = 0.050, β = 0.016, 95% CI: −0.089–0.109, *p* = 0.842), or NI-TBUT (B = 0.038, SE = 0.064, β = 0.049, 95% CI: −0.090–0.165, *p* = 0.560).

To address the potential role of ocular biometry, models incorporating AL were also evaluated (Supplementary Table S9). When SE and AL were entered simultaneously, severe multicollinearity was observed VIF > 18.0, tolerance = 0.054). In separate non-collinear models incorporating AL alongside screen time, age, and sex, AL demonstrated no independent association with OSDI (B = −0.547, *p* = 0.781), TBUT (B = −0.130, *p* = 0.691), or NI-TBUT (B = −0.178, *p* = 0.666).

Multicollinearity was not evident in the final models (Table 6), with tolerance = 0.950 and VIF = 1.052 for both predictors. The maximum condition index was 6.932. Overall, screen time showed the most consistent association with the examined dry eye parameters even after adjustment for SE, age, and sex, whereas SE was not significantly associated with OSDI, TBUT, or NI-TBUT T in the respective multivariable models.

## 4. Discussion

The present study examined the relationships between refractive error severity, AL, digital screen exposure, and DED in young Saudi adults. To our knowledge, few studies have simultaneously evaluated refractive error, AL, digital screen exposure, and both objective and subjective dry eye parameters within a young Middle Eastern population, making these findings particularly relevant to young populations in the Middle East, where digital device use is increasing and refractive errors are common [8,11,12].

The main finding of this study was that greater digital screen exposure was consistently associated with more severe dry eye symptoms and poorer tear film stability. Although significant unadjusted differences in OSDI and NI-TBUT were observed across specific refractive error groups, neither spherical equivalent nor axial length demonstrated independent association with OSDI, TBUT, or NI-TBUT after adjustment for age, sex and screen time in the multivariable models. These findings suggest that digital screen exposure may be a more important modifiable factor associated with dry eye outcomes than refractive error in this young population.

Significant differences in OSDI and NI-TBUT were observed across the six refractive error groups. Participants with high myopia had the highest mean OSDI scores and lowest NI-TBUT values, whereas the low hyperopic group had the lowest OSDI scores and highest NI-TBUT values. These findings are consistent with previous evidence suggesting that myopia is associated with a greater burden of dry eye symptoms and tear film instability. A recent systematic review and meta-analysis reported significantly higher OSDI scores and shorter tear film breakup times among individuals with myopia compared with non-myopic individuals [24]. Similarly, recent studies have reported higher dry eye symptom scores among individuals with higher degrees of myopia and associations between greater myopia or AL and ocular surface abnormalities [6]. The consistency between these reports and the present findings suggests that dry eye parameters may differ across refractive error categories, particularly with the higher symptom scores and lower NI-TBUT observed in the high-myopia group in the present study.

Axial length showed a weak positive correlation with OSDI scores (r = 0.174, *p* = 0.065), but this association was not statistically significant. Similarly, AL was also not significantly associated with TBUT or NI-TBUT. In multivariable linear regression models adjusted for screen time, age, and sex, AL likewise demonstrated no independent association with OSDI, TBUT, or NI-TBUT (Supplementary Table S9). These findings suggest that AL was not independently associated with subjective dry eye symptoms or tear-film stability in the present population. Previous studies have reported associations between greater AL and dry eye symptoms or tear film instability [6,25], whereas other studies have found no significant relationship between AL and dry eye [5,7]. This variability may reflect differences in age, gender, refractive characteristics, sample size, and assessment methods across studies. Potential mechanisms linking axial elongation with ocular surface changes remain speculative, and were not directly evaluated in the present study.

The strongest and most consistent associations were observed for digital screen exposure. Participants reported an average daily screen exposure of approximately 9.40 h. Screen time was positively correlated with OSDI scores (r = 0.650, *p* < 0.001) and negatively correlated with TBUT (r = −0.585, *p* < 0.001) and NI-TBUT (r = −0.509, *p* < 0.001). In addition, screen time was significantly associated with dry eye severity based on Spearman’s correlation (ρ = 0.639, *p* < 0.001). These findings are consistent with previous evidence linking prolonged digital device use with increased dry eye symptoms and reduced tear film stability [9,26,27,28].

The multivariable analyses further support the importance of screen exposure. Screen time remained significantly associated with OSDI, TBUT, and NI-TBUT after adjustment for spherical equivalent, whereas spherical equivalent, axial length, age and sex were not independently significant in any of the models. The models explained 42.6% of the variance in OSDI, 34.2% in TBUT, and 26.1% in NI-TBUT. These results indicate that screen exposure was consistently associated with both subjective symptoms and objective tear film parameters, whereas spherical equivalent was not independently associated with the examined dry eye outcomes after adjustment.

OSDI scores were also strongly correlated with objective tear film measurements, showing negative correlations with TBUT (r = −0.824, *p* < 0.001) and NI-TBUT (r = −0.737, *p* < 0.001). This indicates that participants reporting greater dry eye symptoms generally demonstrated poorer tear film stability. The consistency between subjective and objective measures strengthens the evidence that the observed screen time associations were reflected in both symptoms and clinical tear film measurements.

The regional context may also be relevant. The hot and dry climate of Saudi Arabia and increasing dependence on digital devices may represent additional environmental and behavioral factors affecting ocular surface health. However, these factors were not directly measured in the present study, and therefore cannot be established as contributors to the observed associations. Future studies should assess environmental conditions and specific screen-use behaviors, including continuous viewing duration and blinking patterns, to determine their independent effects.

Overall, the present findings demonstrate that greater digital screen exposure was consistently associated with increased dry eye symptoms, reduced tear film stability, and greater dry eye severity. Although unadjusted differences in OSDI and NI-TBUT were observed across specific refractive error groups, neither spherical equivalent nor axial length was independently associated with clinical dry eye parameters in multivariable models. These findings highlight the potential importance of modifiable digital use behaviors in the assessment of DED among young adults.

### Strengths and Limitations

A major strength of this study was the simultaneous assessment of refractive error, AL, digital screen exposure, and both subjective and objective dry eye measures in a young Saudi population. However, several limitations should be considered. The single-center, cross-sectional design limits generalizability and prevents causal inference. Refractive error was assessed using non-cycloplegic autorefraction without subjective refraction, which may have resulted in some degree of refractive status misclassification. In addition, the study did not include an emmetropic reference group; therefore, the findings cannot establish whether dry eye parameters differ between eyes with refractive error and those with normal refractive status. Accordingly, the observed differences should be interpreted as comparisons among the included refractive error categories, rather than as evidence of a difference between refractive error and emmetropia. We acknowledge that the use of self-reported screen time may be subject to recall and reporting bias, and may not accurately capture variations in use across weekdays and weekends or distinguish occupational/educational from recreational exposure. In addition, potentially relevant environmental and behavioral factors, including ambient humidity, air conditioning exposure, blink rate, continuous screen use duration, viewing distance, and device type, were not measured. Finally, the findings regarding AL should be interpreted cautiously because no statistically significant associations were observed with OSDI, TBUT, or NI-TBUT. Larger multicenter longitudinal studies incorporating these potential confounders are needed to clarify the independent relationships between refractive characteristics, digital screen exposure, and DED.

## 5. Conclusions

Among the refractive error categories included in this study, participants with high myopia had greater subjective dry eye symptoms and shorter NI-TBUT compared with selected lower refractive-error groups. However, TBUT did not differ significantly across the refractive error groups when the unequal variance assumption was appropriately addressed. Prolonged digital screen exposure was also strongly associated with the examined dry eye parameters, and showed a more consistent association with dry eye symptoms and tear film stability than refractive status. These findings suggest that, although unadjusted differences were observed across certain refractive error categories, refractive status was not independently associated with the examined dry eye outcomes after adjustment for screen time and demographic factors. Unmeasured factors, including sleep, environmental conditions, near-work habits, viewing behavior, and other behavioral factors, may also have contributed to the observed associations. Further longitudinal studies are warranted to clarify the independent relationships between refractive status, digital screen exposure, and dry eye parameters.

## Figures and Tables

**Figure 1:**
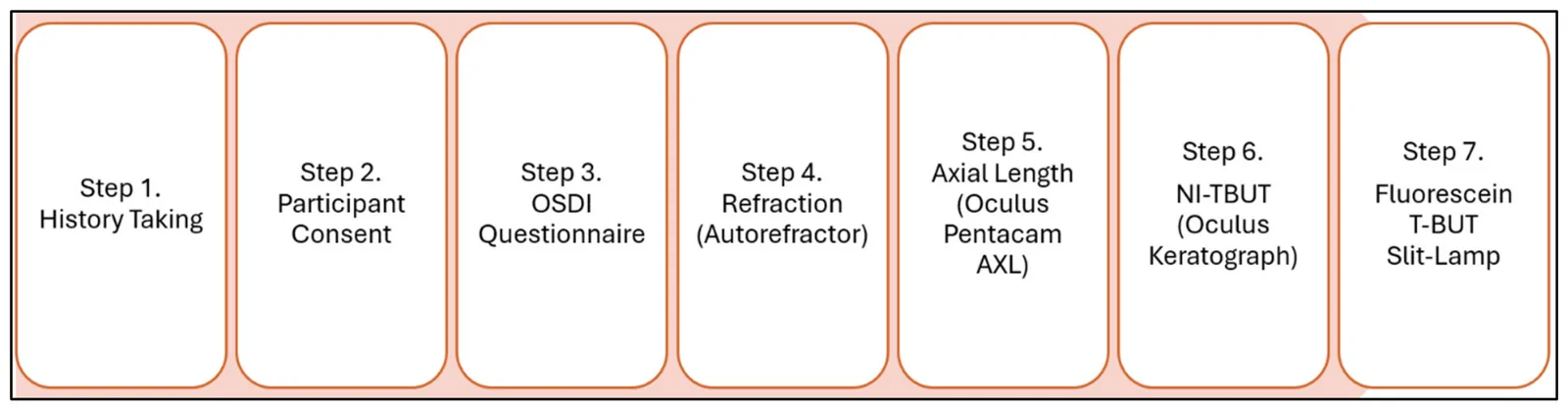
Sequential protocol the study participants underwent.

**Figure 2:**
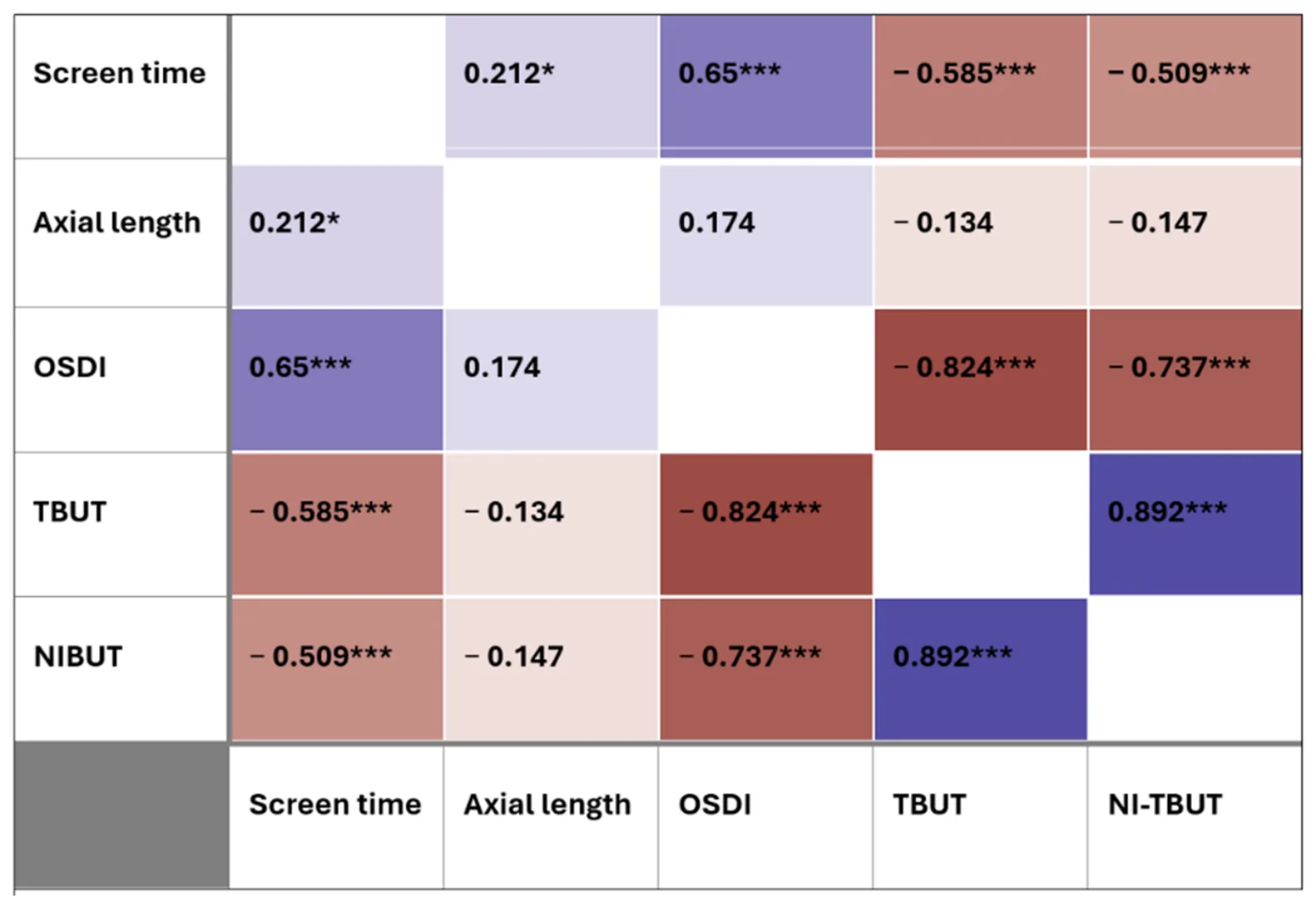
Correlation heatmap among axial length, screen time and dry eye parameters. * (*p* ≤ 0.05), *** (*p* ≤ 0.001).

**Figure 3:**
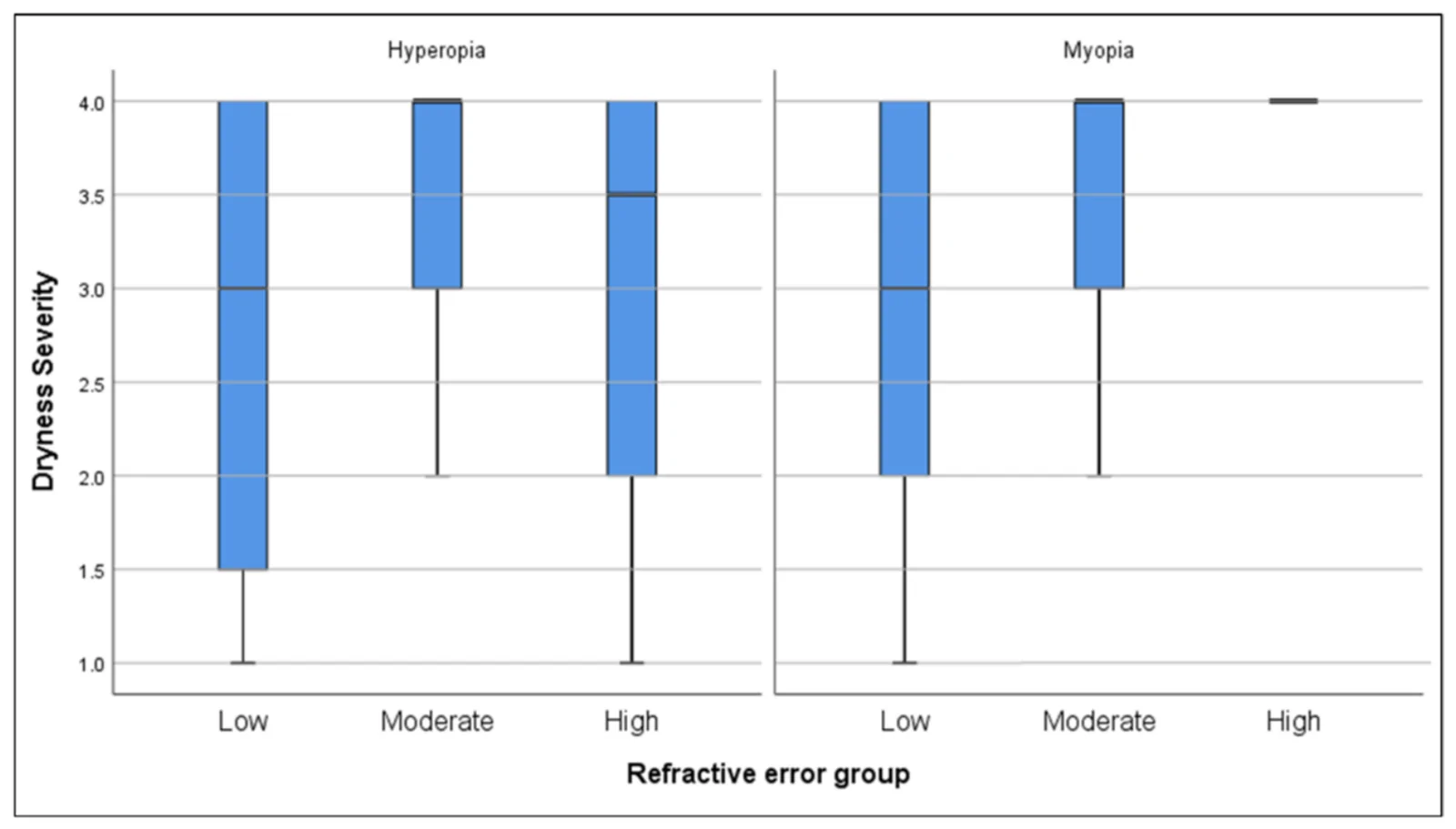
Dryness severity levels by refractive error group. Data are presented as box plots displaying medians, interquartile ranges, and outliers across separate sub-groups for hyperopia and myopia (Low, Moderate, High).

**Table 1:** Degree of refractive error based on spherical equivalent.

Refractive Severity	Spherical Equivalent—SE (D)
High hyperopic	>+5.00
Moderate hyperopic	+2.25 to +5.00
Low hyperopic	+0.50 to +2.00
Low myopic	>−0.50 to −3.00
Moderate myopic	>−3.00 to −6.00
High myopic	>−6.00

**Table 2:** Showing severity of dry eye based on OSDI, NI-TBUT and TBUT.

Dry Eye Severity	NI-TBUT (s)	TBUT (s)	OSDI Score	Interpretation
**Normal**	≥14	>10	0–12	Healthy tear film
**Mild**	11–13	7–10	13–22	Early instability or borderline tear deficiency
**Moderate**	7–10	5–7	23–32	Clinically significant dry eye
**Severe**	<7	<5	33–100	Severe aqueous deficiency and/or instability

The thresholds presented in this table were predefined for the purposes of this study and should not be interpreted as an official TFOS DEWS II composite severity classification. The individual parameters were analyzed separately.

**Table 3:** Baseline characteristics of the study cohort.

Category	Subjects (n)	Mean Age (Years)	Daily Screen Time (h)	Spherical Equivalent (D)	Axial Length (mm)	OSDI Score	TBUT (s)	NI-TBUT (s)
**Myopic**	63	23.63	9.79	−4.24	25.84	37.62	5.11	6.25
**Hyperopic**	51	24.33	9.01	+3.26	21.02	33.67	5.62	7.21
**Total**	114	23.95 ± 3.73	9.40 ± 2.93	−0.88 ± 4.34	23.68 ± 2.70	35.85 ± 18.25	5.34 ± 2.76	6.68 ± 3.33

**Table 4:** The distribution of dry eye severity among the 114 study participants.

Composite Dry Eye	Frequency	Percent
Normal/No Dry Eye	9	7.9
Mild	20	17.5
Moderate	25	21.9
Severe	60	52.6
Total	114	100.0

**Table 5:** Dry eye clinical parameters based on the degree of refractive error.

**Refractive Category**	**Subjects (n)**		**OSDI**	**TBUT**		**NI-TBUT**	
			**Mean ± SD**	**Mean ± SD**		**Mean ± SD**	
Low Hyperopic	20		27.25 ± 16.98	6.55 ± 3.34		8.90 ± 3.50	
Moderate Hyperopic	15		40.13 ± 13.64	4.68 ± 1.76		6.31 ± 2.72	
High Hyperopic	16		35.63 ± 19.50	4.21 ± 2.66		5.96 ± 2.71	
Low Myopic	23		30.04 ± 15.29	5.84 ± 3.00		7.07 ± 3.21	
Moderate Myopic	21		36.00 ± 18.70	5.38 ± 2.21		6.67 ± 3.21	
High Myopic	19		48.58 ± 18.42	3.93 ± 2.46		4.78 ± 2.65	
***p*** -value *			**0.003 ^a^**	**0.080 ^b^**		**0.005 ^a^**	
**Post-Hoc Bonferroni-adjusted Correction—Multivariate Analysis**							
**Dependent Variable**	**I**	**J**	**Mean Difference (I–J)**	**Std. Error**	***p*-value**	**95% CI**	
						**Lower**	**Upper**
OSDI	Low hyperopic	High myopic	−21.33 **	5.52	0.003	−37.89	−4.77
	Low myopic	High myopic	−18.54 **	5.34	0.011	−34.56	−2.51
NI-TBUT	Low hyperopic	High myopic	4.11 **	1.01	0.001	1.08	7.14

* a. One-way ANOVA with df (5,108). ** The mean difference is significant at the 0.05 level. b Welch *F*(5,49.202) = 2.110.

**Table 6:** Multivariable models assessing the independent contributions of screen time and spherical equivalent to dry eye parameters.

Outcome	Model Fit	Predictor	*B*	SE	Standardized β	95% CI for B	t	*p*	VIF
**OSDI score**	R^2^ = 0.426; Adjusted R^2^ = 0.416; F(2,111) = 41.186; *p* < 0.001; SEE = 13.951	Screen time	3.98	0.46	0.638	3.068 to 4.892	8.649	<0.001	1.052
		Spherical equivalent	−0.234	0.31	−0.056	−0.848 to 0.381	−0.753	0.453	1.052
**TBUT (s)**	R^2^ = 0.342; Adjusted R^2^ = 0.330; F(2,111) = 28.884; *p* < 0.001; SEE = 2.256	Screen time	−0.548	0.074	−0.581	−0.695 to −0.400	−7.362	<0.001	1.052
		Spherical equivalent	0.01	0.05	0.016	−0.089 to 0.109	0.2	0.842	1.052
**NI-TBUT (s)**	R^2^ = 0.261; Adjusted R^2^ = 0.248; F(2,111) = 19.641; *p* < 0.001; SEE = 2.890	Screen time	−0.567	0.095	−0.498	−0.756 to −0.379	−5.953	<0.001	1.052
		Spherical equivalent	0.038	0.064	0.049	−0.090 to 0.165	0.585	0.56	1.052

Note: B = unstandardized regression coefficient; SE = standard error; β = standardized regression coefficient; CI = confidence interval; SEE = standard error of the estimate (the square root of the residual mean square); VIF = variance inflation factor. Multicollinearity was not evident in the final models (tolerance = 0.950; VIF = 1.052 for both predictors; maximum condition index = 6.932).

## Data Availability

Data available on request.

## Supplementary Materials

The following supporting information can be downloaded at: https://www.mdpi.com/article/10.3390/jcm15197358/s1, Figure S1: Q–Q plots inspection of Normality for OSDI score- Dryness Severity, TBUT, and NIBUT Across Levels of Refractive Error; Table S1: Shapiro-Wilk Tests of Normality for OSDI score- Dryness Severity, TBUT, and NIBUT Across Levels of Refractive Error; Table S2: Pearson Correlations Among Axial Length, Tear Film Parameters, and OSDI Score (N = 114); Table S3: Pearson Correlations Among Screen Time, Tear Film Parameters, and OSDI Score (N = 114); Table S4: Kruskal–Wallis Test Summary for Dryness Severity Across Levels of Myopia; Table S5: Ordinal Logistic Regression Predicting Dryness Severity from Screen Time; Table S6: Spearman’s Correlation Between Daily Screen Time and Dryness Severity; Table S7: Multivariable Models Assessing the Independent Contributions of Screen Time and Spherical Equivalent to Dry Eye Outcomes; Table S8. Multiple linear regression analyses before and after adjustment for age and sex; Table S9. Multiple linear regression analyses before and after adjustment for age and sex.

## Abbreviations

The following abbreviations are used in this manuscript:

DED	Dry Eye Disease
OSDI	Ocular Surface Disease Index
AL	Axial Length
SE	Spherical Equivalent
NI-TBUT	Non-invasive Tear Break-up Time
TBUT	Tear Break-up Time
*r*-value	Coefficient of Correlation
