# Supplementary material for: Association of Refractive Error Severity, Axial Length and Digital Screen Time with Dry Eye Disease in Young Saudi Adults
Source: J Clin Med. 2026 Sep 22;15(19):7358. doi: 10.3390/jcm15197358 (PMC13666426; doi:10.3390/jcm15197358)
Supplement: Supplementary file 1 [file jcm-15-07358-s001.zip › jcm-4493434-supplementary.pdf]

Supplementary Table S1: *Shapiro-Wilk Tests of Normality for OSDI score- Dryness Severity, TBUT, and NIBUT Across Levels of Refractive Error*

Variable	Refractive Error Level	Shapiro-Wilk		
		Statistic	df	Sig.
OSDI score	Low Refractive Error	.893	23	.018
	Moderate Refractive Error	.923	21	.100
	High Refractive Error	.975	19	.866
TBUT	Low Refractive Error	.911	23	.044
	Moderate Refractive Error	.934	21	.163
	High Refractive Error	.762	19	.000
NIBUT	Low Refractive Error	.899	23	.025
	Moderate Refractive Error	.899	21	.033
	High Refractive Error	.850	19	.007

Supplementary Figure S1: *Q–Q plots inspection of Normality for OSDI score- Dryness Severity, TBUT, and NIBUT Across Levels of Refractive Error*

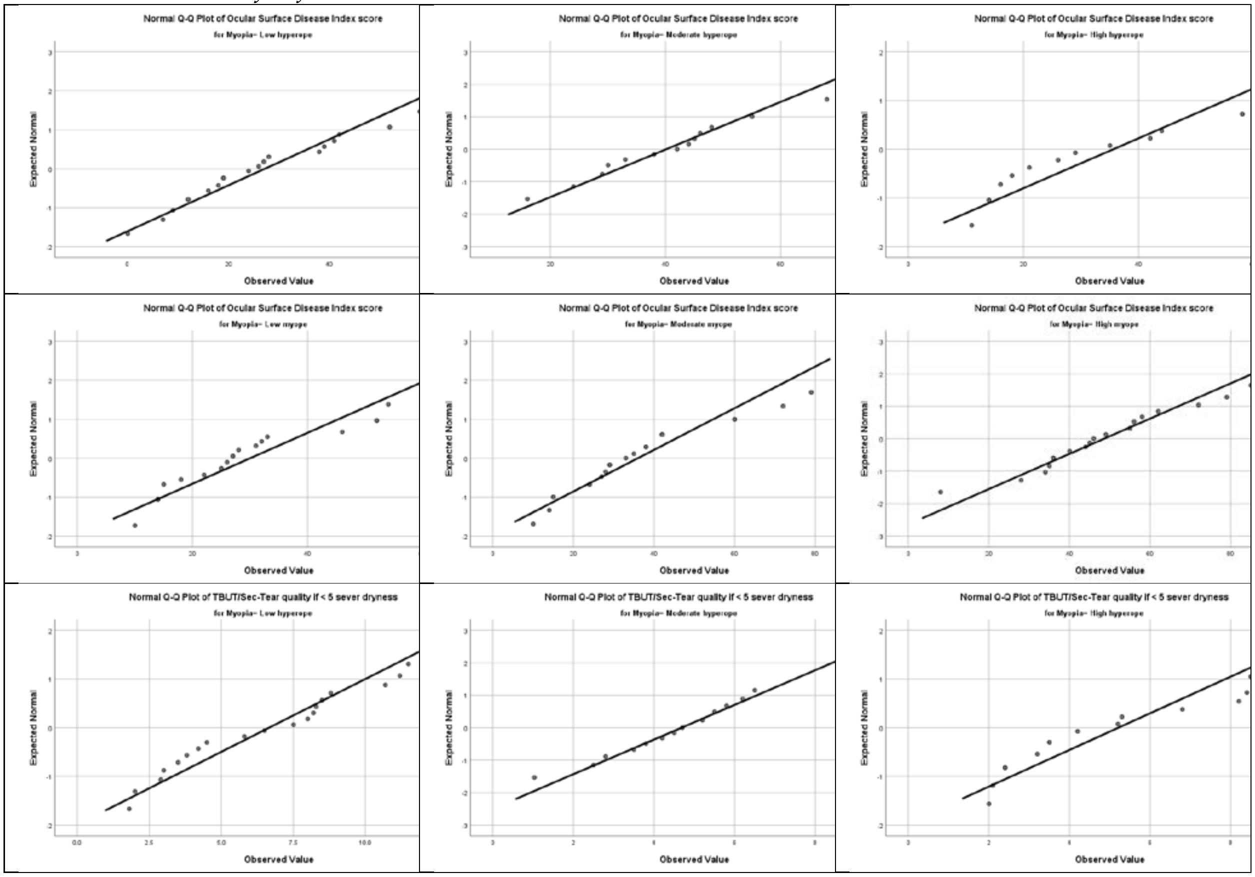

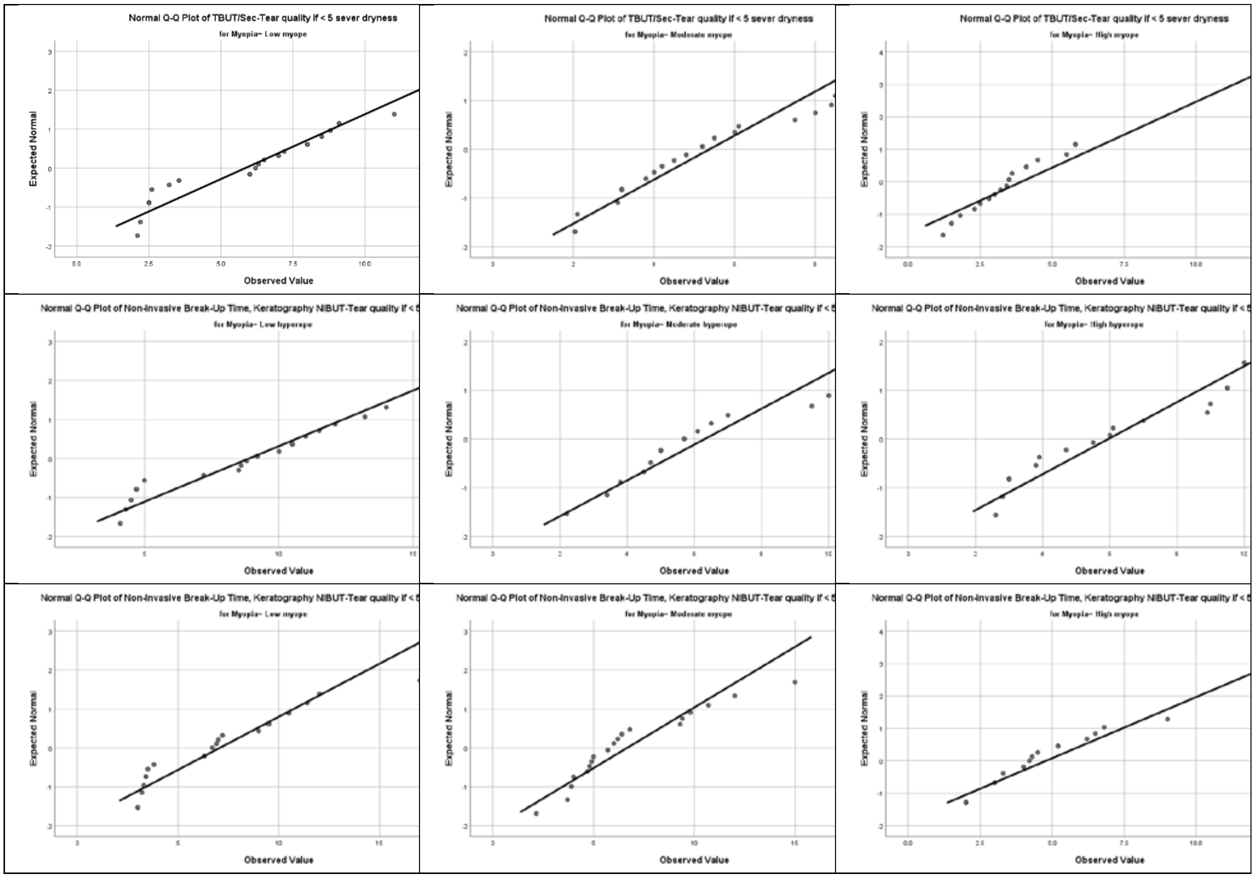

Supplementary Table S2: *Pearson Correlations Among Axial Length, Tear Film Parameters, and OSDI Score (N = 114)*

Variable	Axial length (mm)	TBUT (s)	NIBUT	OSDI score
1. Axial length (mm)		-0.134	-0.147	0.174
2. TBUT (s)			0.892**	-0.824**
3. NIBUT				-0.737**
4. OSDI score				

Note. *TBUT = Tear Break-Up Time, NIBUT = Non-Invasive Break-Up Time, OSDI = Ocular Surface Disease Index. Values are Pearson correlation coefficients (two-tailed).* *$p < 0.05$. ** $p < 0.01$.

Supplementary Table S3: *Pearson Correlations Among Screen Time, Tear Film Parameters, and OSDI Score (N = 114)*

Variable	Screen time (hrs/day)	TBUT (s)	NIBUT	OSDI score
1.Screen time (hrs/day)		-0.585**	-0.509**	0.650**
2. TBUT (s)			0.892**	-0.824**
3. NIBUT				-0.737**
4. OSDI score				

Note. *$p < 0.05$. ** $p < 0.01$.

Supplementary Table S4: *Kruskal–Wallis Test Summary for Dryness Severity Across Levels of Myopia*

Variable	*N*	*H*	*df*	*p*
Dryness Severity	114	18.762	5	0.002

Supplementary Table S5: *Ordinal Logistic Regression Predicting Dryness Severity from Screen Time*

Component	Statistic	Value
Model fit	−2 Log Likelihood, intercept-only	184.824
	−2 Log Likelihood, final model	121.618
	Likelihood-ratio χ^2	63.206
	df	1
	p-value	<0.001
	Nagelkerke R^2	0.47
	McFadden R^2	0.236
	−2 Log Likelihood, intercept-only	0.66
Predictor: Screen time	B	0.095
	Standard Error	48.714
	Wald χ^2	<0.001
	p-value	0.475–0.845
	Odds Ratio, Exp(B)	1.94
	95% CI for OR	1.61–2.33
Proportional-odds assumption	Test of Parallel Lines χ^2	19.302
	df	2
	p-value	<0.001
	Interpretation	Proportional-odds assumption violated

Supplementary Table S6: *Spearman's Correlation Between Daily Screen Time and Dryness Severity*

Variable	Screen time (hrs/day)	Dryness Severity
1.Screen time (hrs/day)		.639**
2. Dryness Severity	.639**	

Note: Spearman's rho correlation coefficient = .639, p < .001, N = 114.
*** Correlation is significant at the 0.01 level (2-tailed).*

Supplementary Table S7: *Multivariable Models Assessing the Independent Contributions of Screen Time and Spherical Equivalent to Dry Eye Outcomes*

Variable	Model fit	Predictor	*B*	SE	Standardized β	95% CI for B	t	*p*	VIF
**OSDI score**	R^2 = 0.426, Adjusted R^2 = 0.416, F(2,111) = 41.186, p < 0.001, SEE = 13.951	Screen time	3.98	0.46	0.638	3.068 to 4.892	8.649	<0.001	1.052
		Spherical equivalent	−0.234	0.31	−0.056	−0.848 to 0.381	−0.753	0.453	1.052
**TBUT (s)**	R^2 = 0.342, Adjusted R^2 = 0.330, F(2,111) =	Screen time	−0.548	0.074	−0.581	−0.695 to −0.400	−7.362	<0.001	1.052

	28.884, $p < 0.001$, SEE = 2.256	Spherical equivalent	0.01	0.05	0.016	−0.089 to 0.109	0.2	0.842	1.052
**NIBUT (s)**	$R^2 = 0.261$, Adjusted $R^2 = 0.248$, $F(2,111) = 19.641$, $p < 0.001$, SEE = 2.890	Screen time	−0.567	0.095	−0.498	−0.756 to −0.379	−5.953	<0.001	1.052
		Spherical equivalent	0.038	0.064	0.049	−0.090 to 0.165	0.585	0.56	1.052

Note: B = unstandardized regression coefficient, SE = standard error, β = standardized regression coefficient, CI = confidence interval, SEE = standard error of the estimate (the square root of the residual mean square), VIF = variance inflation factor. Multicollinearity was not evident in the final models (tolerance = 0.950, VIF = 1.052 for both predictors, maximum condition index = 6.932).

Supplementary Table S8. *Multiple linear regression analyses before and after adjustment for age and sex*

**Variable**	**Model**	**Predictor**	**B**	**β**	**95% CI for B**	***P***
OSDI score	Model 1	Screen time	3.980	0.638	3.068–4.892	<0.001
		Spherical equivalent	−0.234	−0.056	−0.848–0.381	0.453
		$R^2 = 0.426$, Adjusted $R^2 = 0.416$, $F(2,111) = 41.186$, $p < 0.001$, SEE = 13.951				
	Model 2	Screen time	3.931	0.630	3.075–4.786	<0.001
		Spherical equivalent	−0.280	−0.067	−0.859–0.298	0.339
		Age	0.491	0.100	−0.167–1.148	0.142
		Sex	10.019	0.263	4.930–15.108	<0.001
		$R^2 = 0.710$, Adjusted $R^2 = 0.486$, $F(4, 109) = 27.751$, $p < 0.001$, SEE = 13.951				
TBUT (s)	Model 1	Screen time	−0.548	−0.581	−0.695 to −0.400	<0.001
		Spherical equivalent	0.010	0.016	−0.089–0.109	0.842
		$R^2 = 0.342$, Adjusted $R^2 = 0.330$, $F(2,111) = 28.884$, $p < 0.001$, SEE = 2.256				
	Model 2	Screen time	−0.541	−0.574	−0.683 to −0.400	<0.001
		Spherical equivalent	0.012	0.019	−0.084–0.108	0.806
		Age	−0.012	−0.016	−0.120–0.097	0.834
		Sex	−1.458	−0.253	−2.299 to −0.616	0.001
		$R^2 = 0.407$, Adjusted $R^2 = 0.385$, $F(4,109) = 18.673$, $p < 0.001$, SEE = 2.163				
NIBUT (s)	Model 1	Screen time	−0.567	−0.498	−0.756 to −0.379	<0.001
		Spherical equivalent	0.038	0.049	−0.090–0.165	0.560
		$R^2 = 0.261$, Adjusted $R^2 = 0.248$, $F(2,111) = 19.641$, $p < 0.001$, SEE = 2.890				
	Model 2	Screen time	−0.560	−0.491	−0.739 to −0.380	<0.001
		Spherical equivalent	0.033	0.043	−0.089–0.154	0.594
		Age	0.072	0.080	−0.066–0.210	0.305
		Sex	−1.921	−0.276	−2.989 to −0.852	0.001
		$R^2 = 0.345$, Adjusted $R^2 = 0.320$, $F(4,109) = 14.323$, $p < 0.001$, SEE = 2.747				

***Note:** Model 1 includes screen time and SE. Model 2 additionally includes age and sex. B = unstandardized coefficient, β = standardized coefficient, CI = confidence interval, SE = spherical equivalent, OSDI = Ocular Surface Disease Index, TBUT = tear break-up time, NIBUT = non-invasive break-up time, SEE = standard error of the estimate (the square root of the residual mean square).*

Supplementary Table S9. *Multiple linear regression analyses before and after adjustment for age and sex*

Outcome / Model Predictors	B	*SE*	β	t-value	p-value	95% CI for B	VIF
**OSDI Score**							
**Model 1:** Unadjusted (Biometry & Screen Time)							
(Constant)	41.765	47.544	—	0.878	0.382	-52.457, 135.987	—
Screen time (hrs/day)	3.973	0.461	0.637	8.626	< 0.001	3.060, 4.886	1.053
Spherical equivalent (D)	-1.371	1.271	-0.326	-1.079	0.283	-3.890, 1.148	17.645*
Axial length (mm)	-1.884	2.041	-0.278	-0.923	0.358	-5.928, 2.160	17.560*
R^2 = 0.430, Adjusted R^2 = 0.415, F(3, 110) = 27.705, p < 0.001							
**Model 2:** Fully Adjusted (Age & Sex)							
(Constant)	-16.975	47.046	—	-0.361	0.719	-110.228, 76.278	—
Screen time (hrs/day)	3.929	0.433	0.630	9.064	< 0.001	3.070, 4.788	1.053
Spherical equivalent (D)	-0.611	1.224	-0.145	-0.499	0.619	-3.037, 1.815	18.485*
Axial length (mm)	-0.547	1.966	-0.081	-0.278	0.781	-4.444, 3.350	18.399*
Patient Age (years)	0.496	0.334	0.101	1.486	0.140	-0.166, 1.158	1.013
Patient Sex (1 = Male, 2 = Female)	9.869	2.635	0.259	3.745	< 0.001	4.646, 15.092	1.045
R^2 = 0.505, Adjusted R^2 = 0.482, F(5, 108) = 22.028, p < 0.001, Δ R^2 = 0.075, ΔF(2, 108) = 8.128, p = 0.001							
**TBUT (seconds)**							
**Model 1**: Unadjusted (Biometry & Screen Time)							
(Constant)	8.307	7.716	—	1.077	0.284	-6.985, 23.599	—
Screen time (hrs/day)	-0.548	0.075	-0.581	-7.325	< 0.001	-0.696, -0.400	1.053
Spherical equivalent (D)	0.068	0.206	0.106	0.328	0.744	-0.341, 0.477	17.645*
Axial length (mm)	0.095	0.331	0.093	0.288	0.774	-0.561, 0.751	17.560*
R^2 = 0.343, Adjusted R^2 = 0.325, F(3, 110) = 19.125, p < 0.001							
**Model 2:** Fully Adjusted (Age & Sex)							
(Constant)	16.169	7.776	—	2.079	0.040	0.756, 31.582	—
Screen time (hrs/day)	-0.542	0.072	-0.575	-7.562	< 0.001	-0.684, -0.400	1.053
Spherical equivalent (D)	-0.066	0.202	-0.105	-0.328	0.743	-0.467, 0.334	18.485*
Axial length (mm)	-0.130	0.325	-0.127	-0.399	0.691	-0.774, 0.514	18.399*
Patient Age (years)	-0.010	0.055	-0.014	-0.185	0.854	-0.119, 0.099	1.013
Patient Sex (1 = Male, 2 = Female)	-1.493	0.436	-0.260	-3.429	0.001	-2.356, -0.629	1.045
R^2 = 0.407, Adjusted R^2 = 0.380, F(5, 108) = 14.855, p < 0.001, Δ R^2 = 0.065, ΔF(2, 108) = 5.897, p = 0.004							

Outcome / Model Predictors	B	*SE*	β	t-value	p-value	95% CI for B	VIF
**NIBUT (seconds)**							
**Model 1:** Unadjusted (Biometry & Screen Time)							
(Constant)	8.625	9.880	—	0.873	0.385	-10.956, 28.206	—
Screen time (hrs/day)	-0.567	0.096	-0.498	-5.922	< 0.001	-0.757, -0.377	1.053
Spherical equivalent (D)	0.127	0.264	0.166	0.482	0.631	-0.396, 0.650	17.645*
Axial length (mm)	0.149	0.424	0.120	0.350	0.727	-0.691, 0.989	17.560*
R^2 = 0.262, Adjusted R^2 = 0.242, F(3, 110) = 13.031, p < 0.001							
**Model 2:** Fully Adjusted (Age & Sex)							
(Constant)	17.615	9.876	—	1.784	0.077	-1.961, 37.191	—
Screen time (hrs/day)	-0.560	0.091	-0.492	-6.158	< 0.001	-0.740, -0.380	1.053
Spherical equivalent (D)	-0.075	0.257	-0.098	-0.292	0.771	-0.584, 0.434	18.485*
Axial length (mm)	-0.178	0.413	-0.144	-0.433	0.666	-0.996, 0.640	18.399*
Patient Age (years)	0.074	0.070	0.082	1.051	0.296	-0.065, 0.213	1.013
Patient Sex (1 = Male, 2 = Female)	-1.970	0.553	-0.283	-3.561	0.001	-3.067, -0.874	1.045
R^2 = 0.346, Adjusted R^2 = 0.315, F(5, 108) = 11.410, p < 0.001, Δ R^2 = 0.083, ΔF(2, 108) = 6.887, p = 0.002							

Note. B = unstandardized regression coefficient, SE = standard error, β = standardized regression coefficient, OSDI: Ocular Surface Disease Index, TBUT: Tear Break-Up Time, NIBUT: Non-Invasive Break-Up Time, CI: Confidence Interval, VIF: Variance Inflation Factor.

** Indicates severe multicollinearity (VIF > 10, Condition Index > 30) resulting from entering Spherical Equivalent and Axial Length simultaneously into the regression model.*
